# Intravitreal injection of Bevacizumab in diabetic macular edema

**DOI:** 10.12669/pjms.306.5738

**Published:** 2014

**Authors:** Asim Ateeq, Muhammad Ali Tahir, Alyscia Cheema, Arif Dahri, Saifullah Tareen

**Affiliations:** 1 Dr. Asim Ateeq, FCPS, Department of Ophthalmology, Jinnah Post Graduate Medical Centre (JPMC), Karachi, Pakistan.; 2 Dr. Muhammad Ali Tahir, FCPS, Department of Ophthalmology, Jinnah Post Graduate Medical Centre (JPMC), Karachi, Pakistan.; 3 Dr. Alyscia Cheema, FCPS, FRCS, Department of Ophthalmology, Jinnah Post Graduate Medical Centre (JPMC), Karachi, Pakistan.; 4 Dr. Arif Dahri, FCPS, Department of Ophthalmology, Jinnah Post Graduate Medical Centre (JPMC), Karachi, Pakistan.; 5 Dr. Saifullah Tareen, FCPS, Department of Ophthalmology, Jinnah Post Graduate Medical Centre (JPMC), Karachi, Pakistan.

**Keywords:** Diabetic macular edema (DME), Vascular Endothelial Growth Factor (VEGF), Bevacizumab, Macular thickness

## Abstract

***Objective: ***To assess the effectiveness of intravitreal injection of Bevacizumab in the treatment of diabetic macular edema.

***Methods:*** This case series was conducted at Department of Ophthalmology, Jinnah Post Graduate Medical Centre (JPMC), Karachi. The duration of study was six months from May 26, 2011 to November 25, 2011. The study group comprised of 54 patients of the Diabetic Macular Edema (DME). Intravitreal injection of 1.25 mg of bevacizumab (Avastin) was injected 3.5 mm from the limbus under topical anaesthetic drops. Post procedure follow up was scheduled on 1^st^ post procedure day and after one month. Post procedure Optical Coherence tomography (OCT) was performed in all patients 1 week before and 1^st^ month after 1^st^ injection. The results were statistically analyzed through SPSS 17.

***Results:*** Out of the 54 Eyes of 54 Patients who were given the Intravitreal injection of Avastin (Bevacizumab), 43 Eyes (79.6%) showed more than ten percent decrease in macular thickness from pre-injection thickness, 10 Eyes (18.5%) showed less than ten percent decrease and 1 Eye (1.9%) showed increase in macular thickness post operatively after one month.

***Conclusions: ***Intravitreal injection of Bevacizumab (Avastin) is effective in the treatment of diabetic macular edema.

## INTRODUCTION

Diabetic macular edema is one of the major causes of loss of central vision and visual acuity in diabetic patients. Macular edema is defined as retinal edema and hard exudates within 500 micrometers of the centre of macula, retinal edema one disc diameter or larger, any part of which is within one disc diameter of centre of macula.^1^

Up to 10% of all patients with diabetes will develop diabetic macular edema (DME) during their lifetime.^2^ Vascular endothelial growth factor (VEGF) has been shown to be a critical stimulus in the pathogenesis of macular edema secondary to diabetes.^3^ Diabetic macular edema commonly leads to visual loss in patients with diabetes mellitus and this loss of vision can be irreversible.^4^ Increase in vascular permeability due to diabetes results in leakage of fluid and plasma constituents into the retina leading to DME. So far the treatment of choice in clinically significant macular edema is focal laser photocoagulation. The Early Treatment Diabetic Retinopathy Study (ETDRS) reveals that focal laser photocoagulation plays a beneficial role and reduces the rate of moderate visual loss by 50%.^5^ Patients having media opacity like cataract at times make it impossible for the doctor to do complete macular grid laser, so there is a need for an alternative or adjunctive treatment. Vascular endothelial growth factor (VEGF) was discovered in 1989.^6^ Hypoxia stimulates the normal retinal pigment epithelial cells to secrete VEGF.^7^^,^^8^ Eyes having diabetic macular edema have significantly elevated levels of VEGF.^9^ It is observed that eyes with heavy macular leakage have significantly higher VEGF concentration as compared to eyes with less leakage.^10^ Therefore anti-VEGF treatments can be considered as an adjunctive treatment for DME.^11^ Bevacizumab is a full-length antibody that inhibits all isoforms of the VEGF-A family.^12^ It is licensed by Food and drug administration FDA for the treatment of colorectal carcinoma.^13^

Our objective was^ t^o assess the effectiveness of intravitreal injection of Bevacizumab in the treatment of diabetic macular edema.

## METHODS

The study group comprised of 54 eyes of 54 patients of the Diabetic Macular Edema (DME). The study was approved by hospital ethical review committee. Patients having either gender, aging between 25 to 75 years with any type, duration, level of control and severity of diabetes mellitus and having diabetic macular edema were included (DME). DME was defined as retinal edema or hard exudates within 500micrometers of the centre of macula, retinal edema one disc diameter or larger, any part of which was within one disc diameter of centre of macula as evaluated on OCT. Patients having bleeding disorder, active ocular infection, previous history of intravitrealbevacizumab (Avastin), recent myocardial infarction, uncontrolled hypertension, pregnancy and previous history of focal or grid laser were excluded.

All patients who fulfill the inclusion criteria were selected through diabetic retina clinic, department of Ophthalmology, Jinnah Postgraduate Medical Centre. After the explanation of purpose and procedure of study, informed consent was taken. Proformas were filled. Baseline ocular examination was done. OCT was done on Topcon 3D OCT 2000(picture angle 45 degrees with an in depth resolution of 5 micrometer) one week prior to procedure to assess the macular thickness. Intravitreal injection of 1.25 mg/0.05ml of Bevacizumab (Avastin) was injected 3.5-4mm from limbus under local anaesthesia by researcher. Post procedure OCT was done on all patients 1^st^ month after injection and this was when outcome was determined on the basis of change in macular thickness (as mentioned in operational definition). OCT was carried out to ensure effectiveness of the aforementioned medicine for diabetic macular edema. Final outcome was determined on the basis of thickness of macula. Being the Federal Government Hospital, all patients were studied and the funding was borne by the government. Data was analysed using SPSS version 17. 

Descriptive statistics like mean, SD were calculated for age and duration of diabetes mellitus. Frequencies and percentages were calculated for categorical variables like gender, type of diabetes and severity of disease, diabetic control and reduction in macular thickness.

## RESULTS

Within the six months period, total of 54 Patients were selected for this study. Intravitreal Injection of Bevacizumab was given in 54 Eyes of above mentioned 54 Patients. Out of the 54 Patients, 31 were males and 23 were females. Ages of patients ranged from 29 to 71 years (mean 55.44 ± 7.386 years. The mean duration of diabetes was 10.15 years ± 3.21 years. 10 Eyes were of Insulin dependent diabetic (IDDM) patients whereas 44 Eyes were of Non insulin dependent diabetic (NIDDM) patients.

Out of the 54 Eyes treated for this study, 34 Eyes (63%) were of patients in whom diabetes was controlled on medicines whereas 20 Eyes (37%) were of patients whose diabetes was uncontrolled through medicines.

Mean Pre Avastin macular thickness was 384.38 ± 40.51 micrometers and post Avastin OCT after one month showed mean thickness of 323.19±32.58micrometers. Macular thickness after one month of injection was decreased in 53 cases and increased in one case. In 43 patients (79.6%) out of 54 macular thickness decreased significantly i.e. more than 10% and in 10 patients (18.5%) decrease in macular thickness was less than 10% however in 1 patient (1.9%) macular thickness increased.

## DISCUSSION

In patients with media opacity such as vitreous hemorrhage or cataract, it is not always possible to administer complete grid laser. Furthermore, patients with iris neovascularization and neovascular glaucoma often present with hyphema or corneal edema, which prevent full laser treatment, and there are cases that despite grid laser some patients continue to have increased macular thickness (edema). However, Intravitreal Bivacizumab has shown dramatic and rapid response and is found to be effective with complete resolution of macular edema within days. A total of 54 Patients were selected for this study. Intravitreal Injection of Bevacizumab was given in 54 Eyes of above mentioned 54 Patients. Out of them 31 were males and 23 were females. Their ages ranged from 29 to 71 years with a mean of 55.44 ± 7.38 years. The maximum numbers of patients were in their fifth decade. Since the study was done in a government hospital, larger numbers of our patients were from poor financial background. This could be attributed to their poor diabetic control, comparatively lesser attention towards health care issues and compliance towards follow ups. The attributing factor towards this age group was longer duration of diabetes and related complications. However in a similar study conducted by Mason et al, ages of the 30 patients ranged from 26 to 63 years with a mean of 47.7 **±** 12.5 years.^14^ In another study conducted by Avery et al., ages of 32 x patients ranged from 27 to 82 years with mean age of 58 years.^15^ Arevalo JF et al. in a study reported mean age of 57.2 years (range from 23 to 82 years) out of 33 consecutive patients (44 eyes).^16^ The studies on diabetic retinopathy patients showed variation in the age groups affected in different geographical settings.

**Table-I:** Reduction in Macular thickness following single intravitreal injection of Avastin at one month follow up.

**Reduction in Macular Thickness**	**Frequency**	**Percentage**
Yes > 10%	43	79.6
Yes < 10%	10	18.5
Increased	1	1.9
Total	54	100

**Fig.1A:**
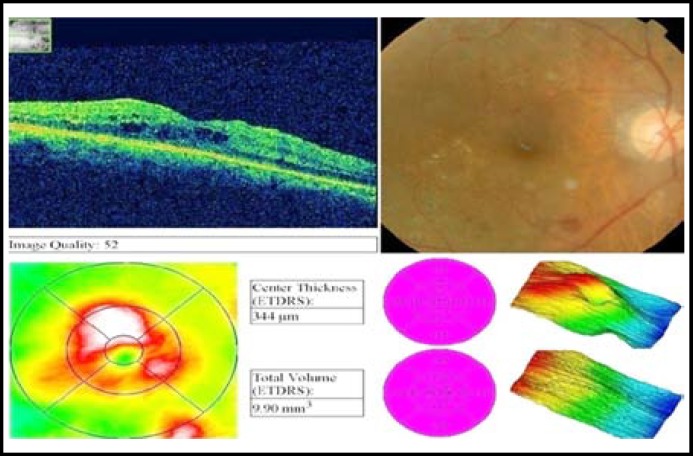
Pre avastin OCT of a patient.

**Fig.1B:**
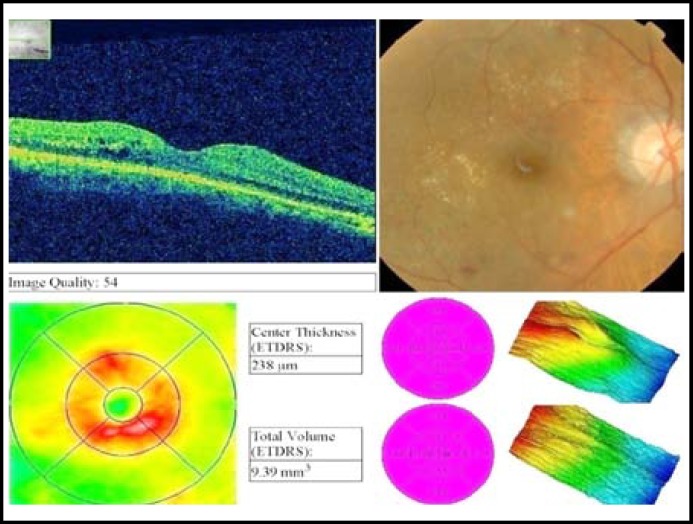
Post avastin OCT of same patient after one month.

**Fig.2A:**
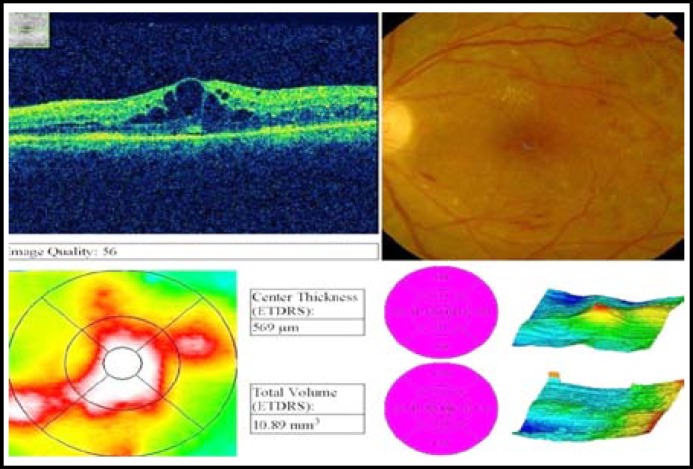
Pre avastin OCT of a patient.

**Fig.2B:**
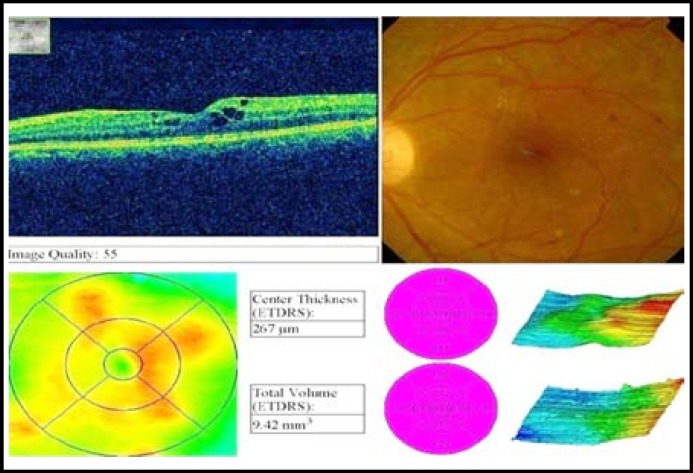
Post avastin OCT of a patient after one month.

In a study conducted by El Haddad et al., age of the patient was also related to the occurrence of retinopathy; however due to logistic model it lost its significance after adjusting for the duration of diabetes and it seemed that it was coupled to the duration of diabetes and could not be regarded as an independent risk factor.^17^ The mean duration of diabetes in our study was 10.15 **± **3.2 years with a range of 4 years to 20 years. In a study conducted by Mason et al. the mean duration of diabetes in 30 patients was 18.4 years with a range of 3 years to 27 years.^14^ Longer the duration of diabetes, the higher the prevalence of diabetic maculopathy. Duration of diabetes was still significant in the multiple logistic model for the occurrence of any retinopathy. This may be caused by a bias in estimating the real duration of diabetes in such patients, especially in NIDDM cases where the discovery of diabetes could have been delayed.^18^

Diabetic macular edema is the most common complication in patients with diabetes mellitus and is a major cause of blindness in the population of working age. A number of studies have shown marked difference in the prevalence of diabetic retinopathy whether in IDDM or in the NIDDM. In our study, out of the 54 Eyes of 54 Patients, 10 Eyes (18.5%) were of Insulin dependent diabetic (IDDM) patients whereas 44 Eyes (81.5%) were of Non insulin dependent diabetic (NIDDM) patients. Arevalo et al. conducted a study in which 23 patients (69.7%) out of 33 patients had IDDM whereas 10 patients (31.3%) had NIDDM.^16^ El Haddad in a study considered 212 patients, out of which 176 patients (83%) had NIDDM whereas 36 patients (17%) had IDDM.^17^ Mason et al. found out that out of 30 patients considered for his study, 17 patients had NIDDM, and 13 patients had IDDM.^14^ Out of the 54 Eyes treated for this study, 34 Eyes were of patients whose diabetes was controlled on medicines whereas 20 Eyes were of patients whose diabetes was uncontrolled through medicines. In our study all IDDM patients were well controlled whereas patients with NIDDM showed variable pattern of control of diabetes, out of all 34 (63%) patients were well controlled however 20 (37%) patients had uncontrolled diabetes. In our study mean central macular thickness at baseline before giving intravitreal injection of Bevacizumab (Avastin) was 384.38 **±** 40.51. Minimum central macular thickness was 290.00um and maximum was 510.46 um. Central macular thickness after one month of intravitreal injection of Bevacizumab (Avastin) showed remarkable reduction and mean central macular thickness dropped to 323.19 ± 32.58 um and minimum and maximum values of central macular thickness were 250.78 and 389.76um respectively. Haritoglou et al. published a prospective, noncomparative case series of patients with DME treated with 1.25 mg Bevacizumab. There was a significant reduction in macular thickness at 2 weeks, the central retinal thickness showed a considerable reduction (33%): from 498.96±123.99 µm at baseline to 334.40±121.76 µm at 1 month.^19^ In other study by Arevalo JF et al. published review of the clinical records of 88 consecutive patients (110 eyes) with DME which shows Mean central macular thickness at baseline by OCT was 387.0+/-182.8 mum and decreased to a mean of 275.7+/-108.3 at end of follow-up. No ocular or systemic adverse events were observed.^16^ Mean decrease in central macular thickness after single intravitreal injection of 1.25mg/0.05ml of Bevacizumab noticed after one month was 61.49 ± 33.21um. 43 eyes (79.6%) showed greater than 10% reduction in macular thickness 10 eyes (18.5%) revealed less than 10% reduction in macular thickness and in 1 eye (1.9%) macular thickness increased to 0.34um one month postoperatively.

## CONCLUSION

Even a transient effect of intravitreal bevacizumab may prove to be of benefit in a variety of clinical settings, such as when media opacity prevents the placement of macular grid laser, or in cases of severe proliferative diabetic retinopathy with concurrent macular edema. In this condition injection of bevacizumab in conjunction with panretinal photocoagulation may minimize the exacerbation of macular edema, which can sometimes be caused by panretinal photocoagulation.

## Authors Contribution:

**AS: **Conceived the study and managed OCT’S of all patients pre and post operatively.

**MAT:** Contributed in design of study, acquisition of data, critical review and final approval of manuscript.

**AC: **Contributed in analysis, interpretation, critical review and final approval of manuscript.

**AD: **Contributed in study design and drafting the article and final approval of manuscript.

**ST: **Contributed in literature search and acquisition.

## Notes

***Grant Support and Financial Disclosures: ***None.
